# The Effect of Recombinant Protein Production in *Lactococcus lactis* Transcriptome and Proteome

**DOI:** 10.3390/microorganisms10020267

**Published:** 2022-01-25

**Authors:** Gabriel A. Monteiro, Sofia O. D. Duarte

**Affiliations:** 1 iBB—Institute for Bioengineering and Biosciences, Department of Bioengineering, Instituto Superior Técnico, Universidade de Lisboa, Av. Rovisco Pais, 1049-001 Lisboa, Portugal; gabmonteiro@tecnico.ulisboa.pt; 2 iBB—Institute for Bioengineering and Biosciences, Instituto Superior Técnico, Universidade de Lisboa, Av. Rovisco Pais, 1049-001 Lisboa, Portugal

**Keywords:** recombinant protein production, systems biology, *Lactococcus lactis*, cell factory, transcriptome, proteome

## Abstract

*Lactococcus lactis* is a food-grade, and generally recognized as safe, bacterium, which making it ideal for producing plasmid DNA (pDNA) or recombinant proteins for industrial or pharmaceutical applications. The present paper reviews the major findings from *L. lactis* transcriptome and proteome studies, with an overexpression of native or recombinant proteins. These studies should provide important insights on how to engineer the plasmid vectors and/or the strains in order to achieve high pDNA or recombinant proteins yields, with high quality standards. *L. lactis* harboring high copy numbers of plasmids for DNA vaccines production showed altered proteome profiles, when compared with a smaller copy number plasmid. For live mucosal vaccination applications, the cell-wall anchored antigens had shown more promising results, when compared with intracellular or secreted antigens. However, previous transcriptome and proteome studies demonstrated that engineering *L. lactis* to express membrane proteins, mainly with a eukaryotic background, increases the overall cellular burden. Genome engineering strategies could be used to knockout or overexpress the pinpointed genes, so as to increase the profitability of the process. Studies about the effect of protein overexpression on *Escherichia coli* and *Bacillus subtillis* transcriptome and proteome are also included.

## 1. Introduction

Lactic Acid Bacteria (LAB) are a group of Gram-positive bacteria that produce lactic acid from the fermentation of sugars. They are chemo-organotrophic and non-spore forming bacteria, which can be found in fermented food and beverages, in the intestinal and genital tract of human and animals, and on plants [1]. *Lactococcus lactis* is a non-pathogenic LAB with a wide applicability in industry, especially as a starter for cheese fermentation. This species could also be found in the gastrointestinal tract of animals and on plants’ surfaces. *L. lactis* is also considered a research model in molecular biology and in the metabolism of LAB [2].

The major prokaryotic system used in the microbial cell factory is *Escherichia coli*, since it is able to produce very high levels of plasmids and recombinant proteins. Since several *L. lactis* strains are considered food-grade and Generally Recognized As Safe (GRAS), as opposed to *E. coli*, which produces highly immunogenic lipopolysaccharides (LPS), they are for use as cell factories for biotechnology applications [3,4]. Traditionally, *L lactis* have been used for the production of food- or pharmaceutical-grade proteins or metabolites for industrial application [5]. Additionally, this species has also been used for the production of pharmaceutical-grade plasmid DNA for DNA vaccination [6,7], or as live vectors for the delivery of DNA, proteins or metabolites to mucosal surfaces [8], which contributed to a rise in the industrial (pharmaceutical, food, cosmetic and energy) relevance of this species [9].

With the availability of sequenced genomes and related data from different levels (e.g., genome, transcriptome, proteome, metabolome), together with genome editing tools, systems biology has emerged as an increasingly relevant field for the study of *L. lactis* [10,11]. The first step in gene expression is the transcription of the stored DNA genetic information into mRNA by RNA polymerase. There are several techniques and tools for a transcriptome analysis, such as DNA microarray, quantitative real-time PCR and high throughput sequencing (RNA-seq) [12]. The next step in gene expression is the translation of the mRNA into a protein. Proteomics can help to characterize the cell proteome at a given time by one of the following major groups of techniques: antibodies-based methods, such as ELISA (Enzyme-linked immunosorbent assay), immunoprecipitation, immune-electrophoresis and Western blot; gel-based methods, such as two-dimensional gel electrophoresis and differential gel electrophoresis; chromatographic methods, such as ion-exchange, size exclusion and affinity chromatography; analytical, functional and reverse phase microarrays; mass spectrometry methods; quantitative techniques, such as ICAT (isotope-coded affinity tag labeling), SILAC (stable isotope labeling with amino acids in cell culture) and iTRAQ (isobaric tag for relative and absolute quantitation); X-ray crystallography; and nuclear magnetic-resonance spectroscopy [13].

This review intends to critically analyze the most recent studies dealing with the effect of overproducing recombinant proteins in the transcriptome and proteome of *L. lactis*. The detailed outcomes of the production of cytoplasmic and membrane-bound proteins in *L. lactis* transcriptome and proteome will also be addressed. This information can be used to design and engineer optimized strains for recombinant protein production, enhancing the value of *L. lactis* as a cell factory, by the overexpression of a rate-limiting gene or the deletion of a disadvantageous gene. The transcriptomic and proteomic analysis could also identify the best (or worse) producing clones, as well as the rate-limiting steps in relevant pathways. At a bio-process level, transcriptome and proteome studies can help to define the feeding strategy and optimize the culture conditions (e.g., temperature, pH, aeration) [12]. The cell response in diverse environments under different stresses could also provide important insights into how *L. lactis* cope with heterologous protein expression.

## 2. *L. lactis* Core Genome and Proteome

The comparative genomics analysis of thirty *L. lactis* strains [14] could provide a more representative insight of the species’ pan- and core genomes. The average chromosome length was 2428 Mbp, with a core genome composed of 1129 genes, but a pan-genome with 5906 genes, of which the majority are short hypothetical coding sequences. The COG (Clusters of Orthologous Genes) analysis showed similar results to the one performed by Silva et al. [15], with translation, ribosomal structure and biogenesis being the most abundant biological processes (excluding the proteins with unknown or general function). Amino acid’s transport and metabolism processes are the second most represented COG, followed by transcription, carbohydrate transport and metabolism, replication, recombination and repair, and inorganic ion transport and metabolism. A more recent analysis of 43 *L. lactis* strains showed a larger pan-genome with 7795 orthologous genes, but 11% of which are plasmid-encoded, and a core genome with 1463 genes [16].

A comparative proteomic analysis of four relevant biotechnological *L. lactis* strains (*L. lactis* subsp. *lactis* NCDO2118, *L. lactis* subsp. *lactis* IL1403, *L. lactis* subsp. *cremoris* NZ9000 and *L. lactis* subsp. *cremoris* MG1363) widely used for biotechnology applications, such as plasmid DNA (pDNA) production for DNA vaccination and/or recombinant protein expression for mucosal vaccination, allowed for the characterization of *L. lactis* core proteome [15]. This study showed that the core genome of *L. lactis* has 1673 genes, while the core proteome shows only 586 proteins with the ribosomal complexes and translational machinery being the most representative proteins, with 19 associated proteins. A KEGG (Kyoto Encyclopedia of Genes and Genomes) enrichment analysis was also performed [15] in order to identify the most represented metabolic pathways in wild-type *L. lactis*. The *L. lactis* core proteome was enriched in pathways related with the ribosome (translation, ribosomal structure and biogenesis), followed by the pyruvate metabolism, microbial metabolism in diverse environments (related to the ability to degrade different compounds to use as carbon and energy source, according to environmental availability), pentose phosphate pathway, nucleotide excision repair and glycolysis and gluconeogenesis. Additionally, several expressed membrane proteins and enzymes responsible for the synthesis of the cell wall (e.g., basic membrane protein A, chitinase, exodeoxyribonuclease, pyruvate carboxylase fibronectin-binding protein), are also present on the core proteome, which are associated with the adhesive properties of *L. lactis* relevant to the mucosal vaccination mechanisms and other immunomodulation processes. A COG analysis of the proteins from the core genome revealed that the most abundant biological process (excluding the proteins with unknown or general function) is the one related to translation, ribosomal structure and biogenesis, followed by replication, recombination and repair, amino acid transport and metabolism, nucleotide transport and metabolism, and carbohydrate transport and metabolism [15].

Some metabolic engineering strategies aiming to increase the production of industrially desirable proteins or metabolites have already been developed. Specifically, for pathways highly represented in the *L. lactis* proteome such as the ones involved in the pyruvate metabolism, the strategies usually include gene knockouts and/or the expression of heterologous enzymes. The aim is to increase the productivity of *L. lactis* for compounds such as alanine (used as a food sweetener and for pharmaceutical applications) [17] or diacetyl (used in many dairy products as well as in the wine industry) [18]. Additionally, metabolic pathways based on enzymes that contribute on a smaller scale to the *L. lactis* core proteome could be engineered to increase the production of pharmaceutically valuable compounds, such as folate (vitamin B11) [19,20,21], riboflavin (vitamin B2) [21] and hyaluronic acid (polysaccharide with medical applications) [22]. Recently, Zhu et al. [23] successfully reduced the genome of *L. lactis* NZ9000 strain by 2.8%, via the deletion of nonessential DNA regions using the Cre-*loxP* deletion system, turning it into a faster growing strain, with a higher biomass yield, an increased ATP content and less maintenance demands. All of these improved features make this strain an attractive host for recombinant-protein production. The examples referred to above emphasize the potential of *L. lactis* to produce aditional industrially apealling compounds in a profitable manner, besides just lactic acid, to which systems biology could have a major contribution.

## 3. Transcriptome and Proteome Profiles of *L. lactis* in Response to Natural Stresses

An analysis of the transcriptome and proteome of *L. lactis* when exposed to a low pH and to high levels of lactate and undissociated lactic acid is relevant for any protein-production setting, since these conditions result from the natural bacterial growth and fermentation metabolism. Wu et al. [24] observed that the acid and lactate stresses inhibited the carbohydrate and energy metabolisms and affected the cell growth probably due to the feedback inhibition from lactic acid, together with the up-regulation of the arginine deiminase pathway, as a way to maintain the stability of the intracellular pH. Several molecular chaperones and proteases were differentially regulated as a response to lactic acid stress, while the expression of DNA repair proteins (DnaA, DnaN and LigA) was down-regulated. With increasing concentrations of lactic acid, the expression of the cell wall genes changes concomitantly, with several up- and down-regulated genes, resulting in peptidoglycan hydrolysis and cell autolysis [24].

Wu et al. [25] analyzed several genes that were involved in *L. lactis* resistance to acid stress. A strain overexpressing *ythA* (PspC family transcriptional regulator) had a 3.2-fold higher survival rate in response to a pH 3.0 acid shock, when compared with the wild-type strain. A transcriptome analysis of the strain overexpressing *ythA* showed that it had an up-regulation of the genes involved in the biosynthesis of amino acids, pyrimidines and exopolysaccharides [25]. The overexpression of the genes *arcB* (amino acid metabolism) and *malQ* (carbon metabolism) resulted in higher survival rates when *L. lactis* was exposed to a pH 4.0 acid shock [26]. The authors also performed a high-throughput screening of mutant libraries generated by UV and chemical mutagenesis. The most acid-tolerant strain revealed that the carbohydrate, amino acid and fatty acids metabolisms were the most affected by the acid stress [26].

Although lactic-acid bacteria are widely used as starters for food and beverages fermentations, the strains used are usually wild-type, without any artificial genetic modifications. The LAB wild-type status could reduce the efficiency of introducing some desired modifications using molecular biology tools. For example, if the strain has active coding genes for endonucleases or extracellular nucleases [27], its transformation with a plasmid will be very difficult because plasmids would be degraded. Furthermore, if the goal is the generation of a strain to produce heterologous proteins, the production of proteases [27] from a wild-type strain is undesired. There are several synthetic biology tools that make it possible to re-design and optimize bacteria, by knocking out non-essential genes, or/and overexpressing others, in order to redirect LAB metabolism for high quality plasmid or protein production [28]. Information from transcriptome and proteome studies are of utmost importance to wisely choosing which genes to remove or to overexpress within *L. lactis* strains, in order to improve its pDNA and heterologous protein expression yields.

## 4. Transcriptome and Proteome Profiles of *L. lactis* in Response to Plasmid DNA and Recombinant Protein Production

The attempts to increase pDNA and recombinant protein production in *L. lactis* have a direct repercussion in its transcriptome, proteome and metabolome, when compared with a wild-type strain [29,30,31]. The findings from such studies are extremely useful for the effectiveness of synthetic biology methodologies, wherein a rational engineering approach of the *L. lactis* genome (gene knockout and/or overexpression) would increase/optimize the pDNA and recombinant protein production, in terms of both yield and quality [28]. When overproducing recombinant proteins, the cells have a high gene dosage due to the high plasmid copy number, together with strong promoters, which in combination result in extremely stressful conditions for the cells. The host cell tends to respond with protective reactions, but also negatively impacts the cell metabolism, and consequently the protein yield and quality [32]. Besides plasmid replication, the expression of plasmid-encoded genes, such as the antibiotic resistance genes, contributes to the metabolic burden, which increases even more when a gene of interest is being expressed [33,34].

### 4.1. Effect of the Overexpression of Membrane and Soluble Proteins in the L. lactis Transcriptome and Proteome

**Figure 1:**
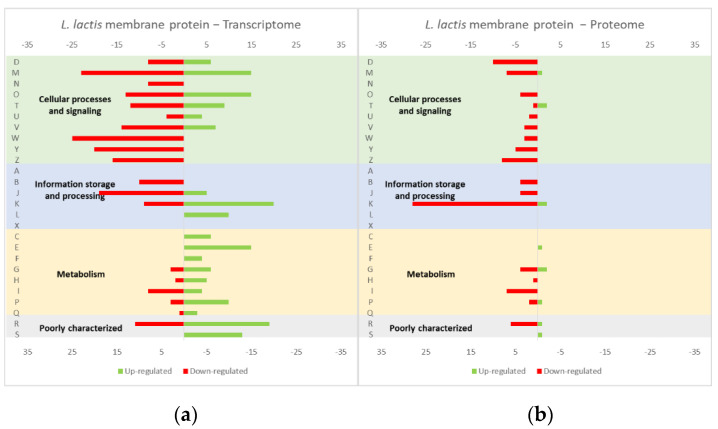
Effect of opuA overexpression in the *L. lactis* (**a**) transcriptome and (**b**) proteome. The graphs were constructed with compiled data from Marreddy et al. [30] and represent the total number of transcripts or proteins up or down-regulated in each cellular process, when compared with the strain harboring the empty vector. The letters on the yy axis correspond to the following COG (Clusters of Orthologous Groups) categories: A, RNA processing and modification (not used for prokaryotic COGs), B, chromatin structure and dynamics, C, energy production and conversion, D, cell cycle control and mitosis, E, amino acid metabolism and transport, F, nucleotide metabolism and transport, G, carbohydrate metabolism and transport, H, coenzyme metabolism, I, lipid metabolism, J, translation, K, transcription, L, replication and repair, M, cell wall/membrane/envelope biogenesis, N, Cell motility, O, post-translational modification, protein turnover, chaperone functions, P, Inorganic ion transport and metabolism, Q, secondary metabolites biosynthesis, transport and catabolism, T, signal transduction, U, intracellular trafficking and secretion, Y, nuclear structure (not applicable to prokaryotic COGs), Z, cytoskeleton (not applicable to prokaryotic COGs); R, general functional prediction only (typically, prediction of biochemical activity), S, function unknown [35,36].

**Figure 2:**
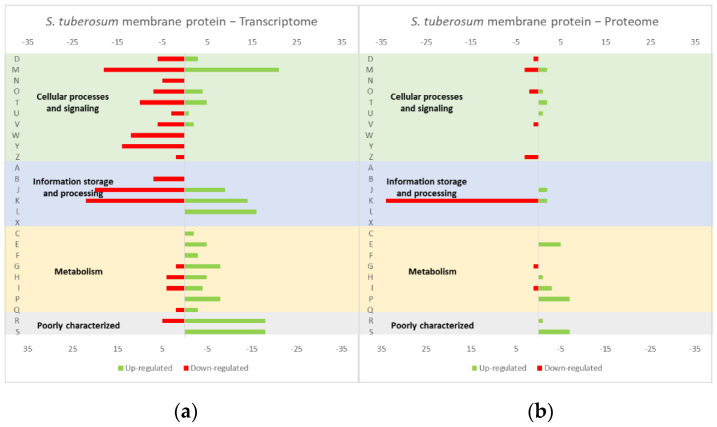
Effect of the StSUT1 expression in the *L. lactis* (**a**) transcriptome and (**b**) proteome. The graphs were constructed with compiled data from Marreddy et al. [30] and represent the total number of transcripts or proteins up or down-regulated in each cellular process, when compared with the strain harboring the empty vector. The letters on the yy axis correspond to the COG categories: A, RNA processing and modification (not used for prokaryotic COGs), B, chromatin structure and dynamics, C, energy production and conversion, D, cell cycle control and mitosis, E, amino acid metabolism and transport, F, nucleotide metabolism and transport, G, carbohydrate metabolism and transport, H, coenzyme metabolism, I, lipid metabolism, J, translation, K, transcription, L, replication and repair, M, cell wall/membrane/envelope biogenesis, N, Cell motility, O, post-translational modification, protein turnover, chaperone functions, P, Inorganic ion transport and metabolism, Q, secondary metabolites biosynthesis, transport and catabolism, T, signal transduction, U, intracellular trafficking and secretion, Y, nuclear structure (not applicable to prokaryotic COGs), Z, cytoskeleton (not applicable to prokaryotic COGs); R, general functional prediction only (typically, prediction of biochemical activity), S, function unknown [35,36].

**Figure 3:**
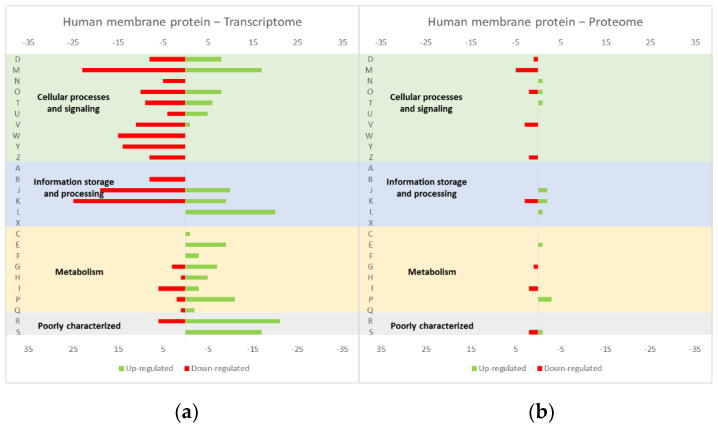
Effect of the PS1Δ9 expression in the *L. lactis* (**a**) transcriptome and (**b**) proteome. The graphs were constructed with compiled data from Marreddy et al. [30] and represent the total number of transcripts or proteins up or down-regulated in each cellular process, when compared with the strain harboring the empty vector. The letters on the yy axis correspond to the COG categories: A, RNA processing and modification (not used for prokaryotic COGs), B, chromatin structure and dynamics, C, energy production and conversion, D, cell cycle control and mitosis, E, amino acid metabolism and transport, F, nucleotide metabolism and transport, G, carbohydrate metabolism and transport, H, coenzyme metabolism, I, lipid metabolism, J, translation, K, transcription, L, replication and repair, M, cell wall/membrane/envelope biogenesis, N, Cell motility, O, post-translational modification, protein turnover, chaperone functions, P, Inorganic ion transport and metabolism, Q, secondary metabolites biosynthesis, transport and catabolism, T, signal transduction, U, intracellular trafficking and secretion, Y, nuclear structure (not applicable to prokaryotic COGs), Z, cytoskeleton (not applicable to prokaryotic COGs); R, general functional prediction only (typically, prediction of biochemical activity), S, function unknown [35,36].

**Figure 4:**
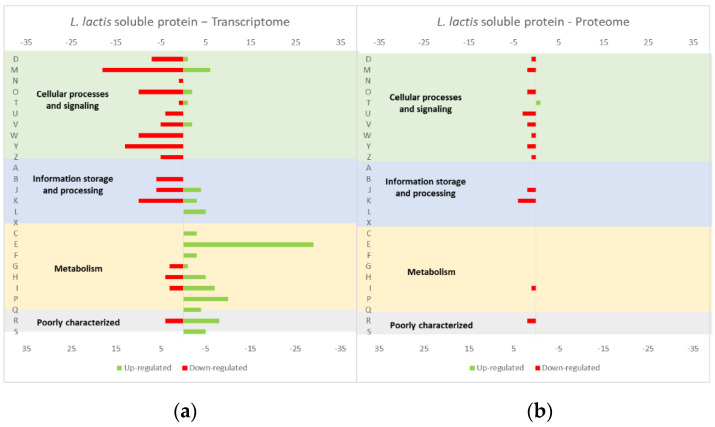
Effect of opuAC (coding for a soluble protein) expression in the *L. lactis* (**a**) transcriptome and (**b**) proteome. The graphs were constructed with compiled data from Marreddy et al. [30] and represent the total number of transcripts or proteins up or down-regulated in each cellular process, when compared with the strain harboring the empty vector. The letters on the yy axis correspond to the following COG categories: A, RNA processing and modification (not used for prokaryotic COGs), B, chromatin structure and dynamics, C, energy production and conversion, D, cell cycle control and mitosis, E, amino acid metabolism and transport, F, nucleotide metabolism and transport, G, carbohydrate metabolism and transport, H, coenzyme metabolism, I, lipid metabolism, J, translation, K, transcription, L, replication and repair, M, cell wall/membrane/envelope biogenesis, N, Cell motility, O, post-translational modification, protein turnover, chaperone functions, P, Inorganic ion transport and metabolism, Q, secondary metabolites biosynthesis, transport and catabolism, T, signal transduction, U, intracellular trafficking and secretion, Y, nuclear structure (not applicable to prokaryotic COGs), Z, cytoskeleton (not applicable to prokaryotic COGs); R, general functional prediction only (typically, prediction of biochemical activity), S, function unknown [35,36].

The studies reviewed below usually include detailed information about the up and down-regulated genes (transcriptome and/or proteome) in response to protein overexpression. These studies generated lots of information that is hard to analyze in terms of raw data. To improve the data analysis capacity and comparability between studies, the genes from each study were compiled into COG categories. For a live mucosal vaccination strategy using *L. lactis*, the expression of an antigen on the cell membrane will make it available for recognition by the host immune system. If one (or more) protein is overexpressed in the cell membrane, the transcriptome and proteome profiles of the *L. lactis* is altered in comparison with the wild-type strain [30]. The overproduction of membrane proteins leads to the up-regulation of several chaperones and proteases, which is consistent with a general stress response to the accumulation of misfolded proteins and, more specifically, with a cell-envelope stress response [30]. Marreddy and colleagues [30] used a lactococcal nisin-inducible gene expression vector (pNZ8048 plasmid) to express several membrane proteins in *L. lactis*, namely the endogenous osmoregulatory ABC transporter OpuA (Figure 1, compiled data from [30]), the plant sucrose transporter StSUT1 from *Solanum tuberosum* (Figure 2, compiled data from [30]) and the human γ-secretase component PS1Δ9 (Figure 3, compiled data from [30]). The effects of the expression of the water-soluble substrate-binding domain OpuAC from *L. lactis* were also compared (Figure 4, compiled data from [30]). The overexpression of the human protein impaired the *L. lactis* specific growth rate the most, followed by the plant gene and the *L. lactis* endogenous gene. Interestingly, the soluble OpuAC-producing cells registered the highest specific growth rate. The mRNA quantification showed that the four genes had similar transcription efficiencies, but the protein expression levels of the prokaryotic genes were one order of magnitude higher than the eukaryotic genes [30].

A more detailed analysis of the up- and down-regulated genes in *L. lactis*, in response to the overexpression of the recombinant protein and compared with the same strain harboring just the empty vector, showed that a majority of transcripts related with cell envelope stress were up-regulated, followed by the general stress response transcripts (Figure 1, Figure 2 and Figure 3, all compiled data from [30]). The stress response was more exacerbated with the expression of the endogenous *opuA* gene than with the eukaryotic genes, since it was produced in much higher amounts. A significant number of transcripts from the CesSR regulon (cell envelope stress response) were also up-regulated, increasing the cell’s ability to remove misfolded proteins and at the same time to correctly fold and insert proteins in the membrane. This stress response was not differently regulated when the endogenous soluble OpuAC protein was overexpressed (Figure 4, compiled data from [30]). Transcripts and proteins related with transcription, protein synthesis and translation were more severely down-regulated following the overexpression of the membrane proteins when compared with the soluble one (Figure 4, compiled data from [30]). The down-regulation of ribosomal proteins is consistent with an increased interaction between ribosomes and the SEC translocon (channel constituted by the SecYEG proteins in the cytoplasmic membrane) and the membrane targeting/insertion process [30]. The overexpression of the membrane recombinant proteins, as opposed to the soluble protein, had a negative impact on the regulation of the transcripts/proteins involved in the synthesis of nucleotides (purines and pyrimidines) via the de novo and salvage pathway, which is probably related with growth impairment. The decreased need for metabolic energy is probably responsible for the down-regulation in the transcripts coding for glycolytic enzymes and pyruvate-dissipating enzymes (carbon and energy metabolism). The genes coding for the enzymes involved in biosynthesis of the peptidoglycan layer were up-regulated both at transcriptome and proteome levels, while the fatty acid synthesis genes were down-regulated [30]. Although, the overproduction of recombinant proteins in *L. lactis* led to more changes at the transcriptome than at the proteome level, whereby the two sets of results pointed to the same overall conclusions. As a consequence of an overall cell-stress response, the expression of recombinant membrane proteins affects house processes, such as transcription, translation, targeting, membrane insertion and folding [30]. Looking at the COG enrichment analysis, the results are consistent, with an up-regulation of the COG E genes (amino acid transport and metabolism) and the down-regulation of the COG M genes (cell wall/membrane/envelope biogenesis) with the overexpression of the soluble protein, while the overexpression of the membrane proteins led to an up- and down-regulation of the COG M genes and the down-regulation of the COG J and K genes (translation, ribosomal structure and biogenesis, and transcription, respectively).

**Figure 5:**
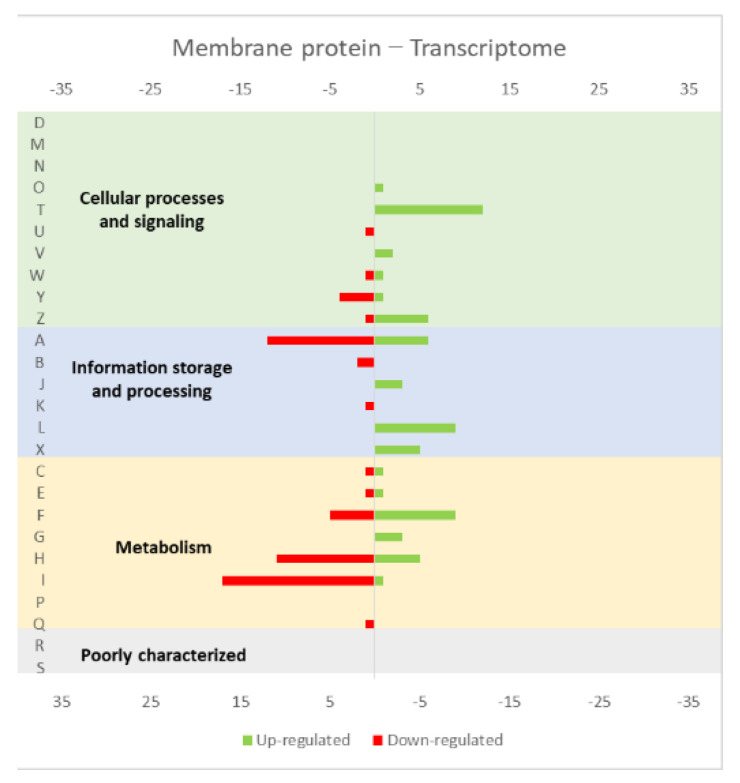
Effect of the membrane proteins overproduction in the *L. lactis* transcriptome. The graphs were constructed with compiled data from Pinto et al. [39] and represent the total number of transcripts up or down-regulated in each cellular process. The letters along the yy axis correspond to the following COG categories: A, RNA processing and modification (not used for prokaryotic COGs), B, chromatin structure and dynamics, C, energy production and conversion, D, cell cycle control and mitosis, E, amino acid metabolism and transport, F, nucleotide metabolism and transport, G, carbohydrate metabolism and transport, H, coenzyme metabolism, I, lipid metabolism, J, translation, K, transcription, L, replication and repair, M, cell wall/membrane/envelope biogenesis, N, Cell motility, O, post-translational modification, protein turnover, chaperone functions, P, Inorganic ion transport and metabolism, Q, secondary metabolites biosynthesis, transport and catabolism, T, signal transduction, U, intracellular trafficking and secretion, Y, nuclear structure (not applicable to prokaryotic COGs), Z, cytoskeleton (not applicable to prokaryotic COGs); R, general functional prediction only (typically, prediction of biochemical activity), S, function unknown [35,36].

Around 30% of the proteome of any organism is constituted by membrane proteins [37] and, in humans, these proteins are the targets of 60% of all pharmaceuticals [38], which increases the relevance of focusing on this protein class. As mentioned above, *L. lactis* is an excellent host for endogenous and heterologous protein production, but membrane proteins have specific constraints of their own, namely their potential hydrophobicity, the difficulty to achieve the correct folding and the low yields of production and purification of the proteins in their native form [39]. It is of utmost importance to analyze the impact of the overproduction of membrane proteins in *L. lactis* in order to try to improve its efficiency and yields, while minimizing negative effects in the microorganism metabolism. Pinto et al. [39] reached similar conclusions to those of Marreddy et al. [30] after analyzing the *L. lactis* transcriptome in response to the overproduction of membrane proteins (Figure 5, compiled data from [39]). Genes from the two-component system CesSR are up-regulated when *L. lactis* is engineered to overproduce membrane proteins. However, this effect is amplified when, instead of an endogenous protein (such as BcaP, a branched-chain amino acid permease), the strain is forced to produce heterologous proteins, such as the eukaryotic presinilin complex. The authors knocked out several genes (*ftsH*, *oxaA2*, *llmg_2163* and *rmaB*) of the CesSR regulon, which resulted in an impairment in the *L. lactis* growth rate and in BcaP protein production. In contrast, the overexpression of the CesSR genes improved the growth rate and both the endogenous and heterologous protein production. The genes from the CesSR regulon seem to be core genes for the overproduction of membrane proteins in *L. lactis*, being characterized as a membrane-protein quality control mechanism by the authors, which allows *L. lactis* to cope with cell envelope damage. There are no reports of CesSR being activated during cytoplasmic protein production or as response to other stresses, such as pH or temperature [39]. Several general stress response genes were also differentially expressed in the strain overproducing BcaP, possibly due to the increase in mis- or unfolded proteins as a result of the overload of the chaperones and translocation machinery. Indeed, the results showed an up-regulation of the genes of the translocation pathway when the BacP protein was produced, but not in the overproduction of the eukaryotic protein, since *L lactis* can only produce trace amounts. The arginine, purine and pyrimidine biosynthetic pathways were down-regulated during BcaP overproduction by *L. lactis*, as well as in transcription and translation [39]. Analyzing these results in terms of COG classification, the strain overproducing the endogenous protein showed an up-regulation of COG T, L and F genes (signal transduction mechanisms; replication, recombination and repair; and nucleotide transport and metabolism, respectively), while the COG I, A and H genes (lipid transport and metabolism; RNA processing and modification; and coenzyme transport and metabolism, respectively) were down-regulated (Figure 5, compiled data from [30]).

### 4.2. Effect of the Growth Rate in the Proteome of L. lactis

**Figure 6:**
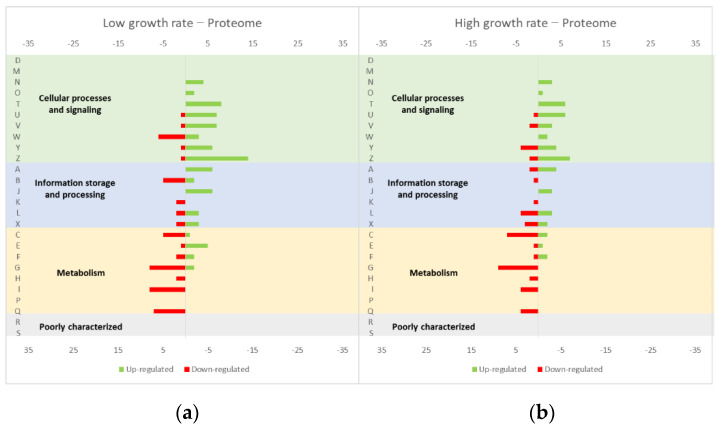
Effect of the growth rate in the *L. lactis* proteome profile: (**a**) low growth rate (0.24 h^−1^) versus (**b**) high growth rate (0.47 h^−1^). The graphs were constructed with compiled data from Dressaire et al. [40] and represent the total number of transcripts up or down-regulated in each cellular process. The letters on the yy axis correspond to the following COG categories: A, RNA processing and modification (not used for prokaryotic COGs), B, chromatin structure and dynamics, C, energy production and conversion, D, cell cycle control and mitosis, E, amino acid metabolism and transport, F, nucleotide metabolism and transport, G, carbohydrate metabolism and transport, H, coenzyme metabolism, I, lipid metabolism, J, translation, K, transcription, L, replication and repair, M, cell wall/membrane/envelope biogenesis, N, Cell motility, O, post-translational modification, protein turnover, chaperone functions, P, Inorganic ion transport and metabolism, Q, secondary metabolites biosynthesis, transport and catabolism, T, signal transduction, U, intracellular trafficking and secretion, Y, nuclear structure (not applicable to prokaryotic COGs), Z, cytoskeleton (not applicable to prokaryotic COGs); R, general functional prediction only (typically, prediction of biochemical activity), S, function unknown [35,36].

Additional interesting data have been found in the work of Dressaire et al. [40], who studied the proteome profile of *L. lactis* IL1403 grown at a steady state in continuous cultures at different growth rates (0.24 and 0.47 h^−1^) (Figure 6, compiled data from [40]). In this case, the strain did not overproduce a protein, but the results could provide important insights about which growth rate is less impairing for the cell metabolism and, consequently, more advantageous for protein production. The authors also compared their data with transcriptomic data previously obtained [41]. Both transcriptomic and proteomic data showed that at high growth rates, the genes related to biogenesis were up-regulated, mainly those genes related to transcription, translation and ribosomal proteins, fatty acid and phospholipid metabolism, cell division, and purine, pyrimidine, nucleoside and nucleotide metabolism. Concerning the stress related proteins, the genes from the two chaperones DnaK and GroEL, the superoxide dismutase related to oxygen stress SodA and the ferritin DpsA, were up-regulated, while CspE (protein associated with cold shock), ClpC (protein associated with heat shock) and Tpx (adaptation related peroxidase) were down-regulated. The levels of other stress-related proteins did not depend on the growth rate, such as ATPases and some peptidases [40]. The authors also tried to establish a relationship between transcriptomic and proteomic data. These two sets of results usually have a weak correlation as a consequence of post-transcriptional regulation (e.g., translation efficiency, protein degradation) [42], protein stability [43,44,45] and protein dilution due to cellular growth. The correlation between transcriptome and proteome data depends on the gene and on growth conditions. At slower growth rates, the protein degradation rate is higher, while translation efficiency is improved [40].

### 4.3. Effect of Plasmid Copy Number in the Proteome of L. lactis

**Figure 7:**
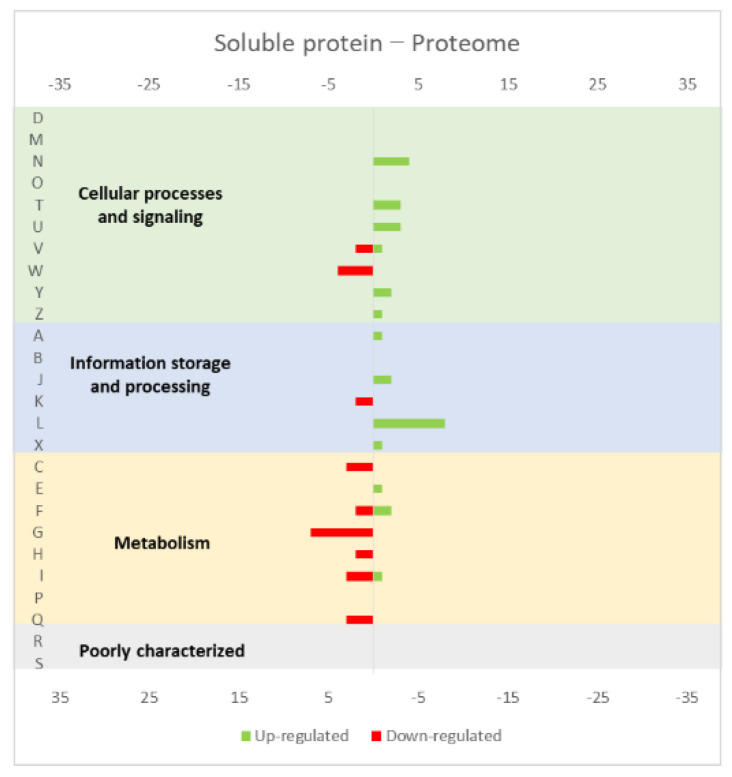
Effect of a heterologous protein overexpression in the *L. lactis* proteome profile. The graphs were constructed with compiled data from Kim et al. [29] and represent the total number of transcripts up or down-regulated in each cellular process. The letters on the yy axis correspond to the following COG categories: A, RNA processing and modification (not used for prokaryotic COGs), B, chromatin structure and dynamics, C, energy production and conversion, D, cell cycle control and mitosis, E, amino acid metabolism and transport, F, nucleotide metabolism and transport, G, carbohydrate metabolism and transport, H, coenzyme metabolism, I, lipid metabolism, J, translation, K, transcription, L, replication and repair, M, cell wall/membrane/envelope biogenesis, N, Cell motility, O, post-translational modification, protein turnover, chaperone functions, P, Inorganic ion transport and metabolism, Q, secondary metabolites biosynthesis, transport and catabolism, T, signal transduction, U, intracellular trafficking and secretion, Y, nuclear structure (not applicable to prokaryotic COGs), Z, cytoskeleton (not applicable to prokaryotic COGs); R, general functional prediction only (typically, prediction of biochemical activity), S, function unknown [35,36].

The overexpression of heterologous proteins by *L. lactis* require cloning of the gene of interest into a plasmid vector with more or less copies per cell and consequently, of the copies of the gene being expressed. It was previously described that the number of plasmid copies per cell (or plasmid copy number, PCN) could influence the expression level of some proteins [46], which in turn leads to alterations in the cell proteome profile. In a recent study (Figure 7, compiled data from [29]), the GFP gene under the control of the strong inducible promoter *nisA* was cloned into the low PCN pHR086 plasmid (6–9 copies per cell) and into the high PCN pJH24 plasmid (45–85 copies per cell), both derived from pIL252 with pAMβ1-based replicons [29]. As a result, the high PCN plasmid expressed 4-fold more GFP than its low PCN counterpart, making GFP the most abundant protein in the bacterial proteome [29]. Several stress response and chaperone proteins (*groES*, *groEL trxB1*, *grpE*, *nusG* and *dnaJ*), as well as ATP synthase, were up-regulated in the high PCN strain to increase protein synthesis and stability, and to allow the cell to cope with the stress of having an increase in the plasmid maintenance costs, while the enzymes of the amino acid pathway (de novo synthesis and interconversion of amino acids) were downregulated [29]. Several glycolytic enzymes and transcription/translation-related proteins were also highly expressed. All these alterations were much more significant when using the high PCN plasmid compared with the low PCN counterpart. A major difference found in the high PCN strain was in the glucose metabolism, with some enzymes belonging to the sugar nucleotide metabolism being up- (*pfl* and *adhE*) or down-regulated (*zwf* and *gnd*—pentose phosphate pathway) [29]. If some pentose phosphate pathway enzymes were down-regulated, the carbon flow for purine and pyrimidine synthesis should have decreased. The overexpression of the GFP protein and probably the increase in the PCN, also altered the expression of pyruvate metabolism enzymes, and decreased the expression of some enzymes from the de novo synthesis pathway for pyrimidine nucleotides towards RNA (*nrdE* and *nrdI*). The expression of some enzymes involved in the cell wall structures also decreases upon GFP overexpression [29]. The COG enrichment analysis showed similar results, with the high PCN strain up-regulating the COG L genes (replication, recombination and repair) and down-regulating the COG G genes (carbohydrate transport and metabolism) (Figure 7, compiled data from [29]).

### 4.4. Insights from E. coli and B. subtilis Studies

The literature referring to changes in the *L. lactis* transcriptome and proteome due to the overexpression of heterologous proteins is scarce, but some inferences can be made from studies with other Gram-positive bacteria [47] and also from the Gram-negative *E. coli* [48,49]. Jϋrgen et al. [47] analyzed modifications in the transcriptome and proteome of *B. subtilis* in response to the overproduction of the insoluble heterologous protein PorA, which accumulates in inclusion bodies when expressed in *B. subtilis*. As was the case with *L. lactis*, several heat-shock genes were up-regulated. The most surprising result was that the genes coding for ribosomal proteins and pyrimidine and purine synthesis enzymes were also up-regulated [47], as opposed to the result observed in *L. lactis*. This result is also not consistent with *E. coli*, where the overexpression of a secreted recombinant protein led to the down-regulation of both the ribosomal proteins and the nucleotide synthesis proteins [48], as observed with *L. lactis*.

Mairhofer et al. [32] performed a study of the changes that occurred in the *E. coli* transcriptome when comparing a plasmid-free with a plasmid-based condition, while overproducing a recombinant protein, which is a type of study that had never been performed on *L. lactis*. In the plasmid-free system, *E. coli* BL21 (DE3) had one copy of the gene of interest (coding for SOD, superoxide dismutase) integrated in the genome, over the control of the T7 promoter, while in the plasmid-based system, the same strain was transformed with a pET30a plasmid harboring the SOD gene. The plasmid-based system showed an up-regulation of chaperones and proteases genes, which should contribute for the correct folding of the recombinant protein, although this did not occurred, since SOD was accumulated in the inclusion bodies. This could indicate that the aggregates sequestered the chaperones and proteases, making them unavailable or impairing the translation of its mRNA into functional proteins. Additionally, the plasmid-based system up-regulated the Psp operon allowing the cell to respond to extracytoplasmic stress, but a down-regulation of the *nuo* operon, indicating an energy shortage. The main conclusion of this study is that a high concentration of the recombinant protein does not impair the host metabolism by itself, rather, the major problem is an increase in the levels of mRNA, which occupies the ribosomes and sequesters amino acids and nucleotides precursors that are necessary for the cell integrity and metabolism [32].

**Figure 8:**
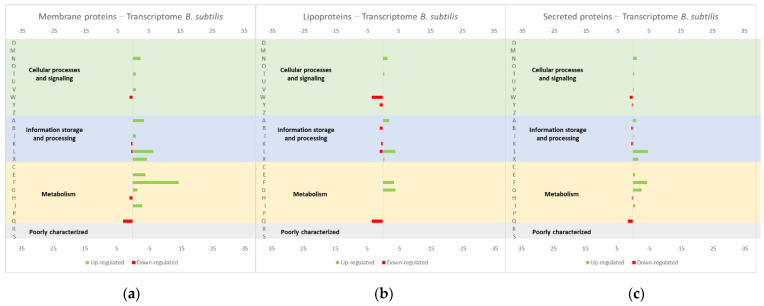
Effect of the overproduction of (**a**) membrane proteins, (**b**) lipoproteins and (**c**) secreted proteins, both endogenous and heterologous, in the *B. subtilis* transcriptome profile. The graphs were constructed with compiled data from Marciniak et al. [50] and represent the total number of transcripts up or down-regulated in each cellular process. The letters on the yy axis correspond to the following COG categories: A, RNA processing and modification (not used for prokaryotic COGs), B, chromatin structure and dynamics, C, energy production and conversion, D, cell cycle control and mitosis, E, amino acid metabolism and transport, F, nucleotide metabolism and transport, G, carbohydrate metabolism and transport, H, coenzyme metabolism, I, lipid metabolism, J, translation, K, transcription, L, replication and repair, M, cell wall/membrane/envelope biogenesis, N, Cell motility, O, post-translational modification, protein turnover, chaperone functions, P, Inorganic ion transport and metabolism, Q, secondary metabolites biosynthesis, transport and catabolism, T, signal transduction, U, intracellular trafficking and secretion, Y, nuclear structure (not applicable to prokaryotic COGs), Z, cytoskeleton (not applicable to prokaryotic COGs); R, general functional prediction only (typically, prediction of biochemical activity), S, function unknown [35,36].

Important insights could also be taken from study [50] about the effect of the overproduction of membrane proteins, lipoproteins and secreted proteins, both endogenous and heterologous, in the *B. subtilis* transcriptome (Figure 8, compiled data from [50]). The studies on the effects of protein overexpression in the *L. lactis* transcriptome and proteome usually focus on membrane or soluble proteins, meaning that important information can be depicted from this study performed in *B. subtilis*. Similar to *L. lactis*, *B. subtilis* is also a Gram-positive bacteria with GRAS properties and a great capability of secreting proteins. Therefore, it is a suitable host for the production of industrially relevant proteins, since it is less prone to forming inclusion bodies, when compared with *E. coli*, but it has a complex extracellular proteolytic system that is responsible for the degradation of secreted recombinant proteins [51]. *L. lactis* have only one protease for secreted proteins [51], being available a protease-free mutant [52]. Additionally, *L. lactis* only secrete one major protein (Usp45), which allows for simpler downstream purification processes. The authors studied the influence of the overproduction of proteins expressed in different cellular locations, namely, membrane proteins (LmrA of *L. lactis* and XylP of *Lactobacillus pentosus*), lipoproteins (MntA and YcdH of *B. subtilis*) and secreted proteins (NprE and XynA of *B. subtilis*, Usp45 of *L. lactis*, TEM-1 β-lactamase of *E. coli*). A general stress response was observed in *B. subtillis* for all of the overexpressed proteins, more specifically with an up-regulation of genes coding for intracellular stress proteins, including *groES*, *groEL* and *CtsR* regulated genes. The overproduction of all secreted proteins resulted in an up-regulation of *cssRS*, *htrA* and *htrB* genes, while only Usp45 and TEM-1 β-lactamase overproduction resulted in the *liaIHGFSR* operon up-regulation. A different response was detected when membrane proteins were overproduced, with an up-regulation of sigW and SigW-regulated genes and *ykrL.* The overproduction of all extracytosolic proteins (the two heterologous secreted proteins and the lipoproteins) induced the CssRS mediated secretion stress response and the up-regulation of the *htrA* and *htrB* genes [50]. Compatible results were obtained by Jürgen [47], wherein *B. subtilis*, overproducing recombinant proteins, showed a transcriptome up-regulation of heat-shock genes of class I (*dnaK*, *groEL*, and *grpE*), class III (*clpP* and *clpC*) and pyrimidine and purine synthesis enzymes.

### 4.5. Effect of Different Stresses in the L. lactis Transcriptome and Proteome

The overproduction of proteins by *L. lactis* can be considered a stress condition, as noted by the overall stress response from the cells. For this reason, studies about the effect of different stresses in the *L. lactis* transcriptome and proteome could provide useful insight into what should be happening during protein overproduction. Van der Meulen et al. [53] studied the effect of cold, heat, acid, osmotic stress and oxidative stress in the *L. lactis* transcriptome, finding that most tRNAs decreased after all stresses. Only a small number of tRNAs increased in response to the cold stress. Concerning other genes, the highest differential response was induced by starvation stress. The changes induced by the majority of stresses were the up-regulation of *pur* and *pyr* operons (de novo synthesis of purines and pyrimidines), the down-regulation of *fruAKR* (response to cell envelope stress), the up-regulation of the *metC-cysK* operon, the up-regulation of genes from the fatty acid biosynthesis pathway (*fab* and *acc*), the up-regulation of protein chaperones (GroEL, GroES, DnaK, DnaJ, and GrpE) and proteases (*clpE*, *clpP,* and *clpB*). There were responses specific to some of the stresses, such as the up-regulation of the *zit* operon (uptake of Zn^2+^) in response to cold stress, or the up-regulation of the transport proteins BusAA-BusAB after osmotic stress [53].

Amino acid-accumulation is a limiting factor for protein overexpression in *L. lactis* [54]. A proteomic analysis of *L. lactis* overexpressing membrane proteins showed a limitation in the availability of branched-chain amino acids and in the bacterial capacity of accumulate them. The authors achieved an increase in protein expression by supplying the cells with alternative paths for the accumulation of Ile, Leu and/or Val, by medium supplementation, or perform genome engineering in *L. lactis* in order to improve the transport capacity for branched-chain amino acids (*bcaP*) [54].

Chen et al. [55] developed RECTA, a computational pipeline for regulon identification based on comparative genomics and a transcriptomics analysis. RECTA was implemented for *L. lactis* MG1363 data in order to discover the regulons involved in the response to the acid stress. The validated acid-response regulatory network included two trans-membrane proteins, eight regulons, nine functional modules and 33 genes with known orthologous. Genome engineering strategies could be used in any of these candidates with the goal of improving acid tolerance. The same rationale could be used to improve plasmid and protein production in *L. lactis*.

## 5. Conclusions

The information from the transcriptome and proteome studies in *L. lactis* provides important insight into methods that can be used to engineer the vectors and/or the strains when the objective is to produce a high quality and quantity of pDNA or recombinant protein for pharmaceutical/industrial applications. One of the most important characteristics to consider when selecting a vector is the type of replication origin, since it will influence the PCN. The PCN in turn affects the *L. lactis* transcriptome and proteome, and ultimately the pDNA and recombinant protein yield and quality. An average PCN vector, such as the pIL253-derived vectors (pAMβ1 replicon), allows for the preservation of the metabolism and integrity of the *L. lactis* cells, representing a good choice for obtaining acceptable amounts of pDNA and protein. Additionally, the promoter strength influences the amount of protein produced and, consequently, impacts the cell metabolism. An alternative could be to engineer a vector (i.e., change the origin of replication, change the promoter) using appropriate synthetic biology tools, for use in the proposed application. The modification of the plasmid vector can only increase its efficacy to a certain extent, making it necessary to also consider the strain modification. The data from how the production of recombinant proteins by *L lactis* affects its transcriptome and proteome could provide important information about the genes available for genome engineering (e.g., knockout or overexpression).

For live mucosal vaccination applications, one should account for the impact on the cell growth and metabolism, since the antigen of interest must be produced during a minimum period of time. The cell-wall anchored antigens, instead of intracellular or secreted ones, demonstrated more promising results in live mucosal vaccination studies. However, the transcriptome and proteome studies show that engineering *L. lactis* to express membrane proteins increases the overall cellular burden.

More studies are needed to investigate ways to engineer the *L. lactis* genome, and consequently, its transcriptome, proteome and metabolome, in order to overcome the hurdles experienced and increase its efficiency and profitability in different applications.
